# Resultant Information Descriptors, Equilibrium States and Ensemble Entropy †

**DOI:** 10.3390/e23040483

**Published:** 2021-04-19

**Authors:** Roman F. Nalewajski

**Affiliations:** Department of Theoretical Chemistry, Jagiellonian University, Gronostajowa 2, 30-387 Cracow, Poland; nalewajs@chemia.uj.edu.pl

**Keywords:** continuity relations, grand ensemble, information sources, phase equalization, reactivity criteria, resultant information

## Abstract

In this article, sources of information in electronic states are reexamined and a need for the resultant measures of the entropy/information content, combining contributions due to probability and phase/current densities, is emphasized. Probability distribution reflects the wavefunction modulus and generates *classical* contributions to Shannon’s global entropy and Fisher’s gradient information. The phase component of molecular states similarly determines their *nonclassical* supplements, due to probability “convection”. The local-energy concept is used to examine the *phase* equalization in the equilibrium, *phase*-transformed states. Continuity relations for the wavefunction modulus and phase components are reexamined, the convectional character of the local source of the resultant gradient information is stressed, and latent probability currents in the equilibrium (stationary) quantum states are related to the *horizontal* (“thermodynamic”) phase. The equivalence of the energy and resultant gradient information (kinetic energy) descriptors of chemical processes is stressed. In the *grand*-ensemble description, the reactivity criteria are defined by the populational derivatives of the system average electronic energy. Their entropic analogs, given by the associated derivatives of the overall gradient information, are shown to provide an equivalent set of reactivity indices for describing the charge transfer phenomena.

## 1. Introduction

In this conceptual work we focus on the overall entropy/information content of electronic wavefunctions in the position representation of quantum mechanics (QM). Such quantum states are described by (complex) vectors in the molecular Hilbert space or by their statistical mixtures. Each state vector is characterized by its modulus (“length”) and phase (“orientation”) components in the complex plane. The square of the former determines the classical descriptor of the state probability distribution, while the gradient of the latter generates the density of electronic current and the associated velocity field reflecting the probability *convection*. These physical descriptors summarize different aspects of the state electronic structure: the probability density represents the *static* “structure of being”, while its flux characterizes the *dynamic* “structure of becoming” [1]. Indeed, in the underlying continuity equation for the (sourceless) probability distribution, the divergence of electronic flow, which shapes its time dependence, determines the local outflow of the probability density. The fundamental Schrödinger equation (SE) of QM ultimately determines the *time* evolutions of the state wavefunction itself, its components, and expectation values of all physical observables.

As complementary descriptors of electronic structure and reactivity phenomena, both the modulus and phase parts of molecular states contribute to the overall (resultant) content of their entropy (uncertainty) and information (determinicity) descriptors [2,3,4,5,6,7,8,9]. The need for such generalized information-theoretic (IT) measures of the entropy/information content in electronic states has been emphasized elsewhere [10,11,12,13,14]. Such descriptors combine the *classical* terms due to wavefunction modulus (or probability density), and the *nonclassical* contributions generated by the state phase (or its gradient determining the convection velocity). The overall gradient information, the quantum extension of Fisher’s intrinsic accuracy functional for locality events, then represents the dimensionless measure of the state electronic kinetic energy [2,3,4,5,6,7,8,9,10,11,12,13,14,15]. This proportionality relation between the state resultant information content and the average kinetic energy of electrons ultimately allows applications of the molecular virial theorem [16,17,18,19,20,21,22,23,24,25,26,27,28,29] in an information interpretation of the chemical bond and reactivity phenomena [4,6,9,30].

In principle, all these entropic contributions can be extracted by an experimental removal of the position and momentum uncertainties in the system quantum state [31,32]. For the parametrically specified particle location in the position representation of QM, both the probability distribution and its effective convectional velocity (current-per-particle) are uniquely specified by the system wavefunction. Therefore they both constitute bona fide sources of the information contained in electronic states, fully accessible in the separate position and momentum experiments.

In the stationary bound states, for the sharply specified energy, time-independent probability distribution and purely *time*-dependent phase, the local phase component and probability convection identically vanish. It will be argued, however, that their (*phase*-transformed) *equilibrium* analogs exhibit latent electronic fluxes along probability contours, which do not affect the stationary probability density. These flows are related to the state local (“thermodynamic”) phase component, proportional to the negative logarithm of probability density, for which the *internal* resultant IT descriptor of electronic state vanishes. Therefore, for this equilibrium criterion the average entropy measure in thermodynamic states becomes identical with von Neumann’s entropy [33], the function of *external* state probabilities defining the density operator of the ensemble mixed state.

In this analysis the quantum dynamics and continuity relations for the modulus (probability) and phase (current) degrees-of-freedom of electronic states are reexamined and their contributions to the resultant entropy/information descriptors are identified. The convection character of the net source of resultant gradient information is stressed, and equivalence of the energy and information criteria of chemical reactivity is emphasized. A distinction between classical (probability) and quantum (wavefunction) mappings is briefly discussed and the *convection* velocity of probability “fluid” is used to define fluxes of general physical and information properties. In such an approach, the system electrons thus act as carriers of the property densities. The latent electronic flows in the quantum stationary equilibrium, which do not affect the probability distribution, are also examined in some detail. Their quantum dynamics is examined and related to the “horizontal” phase component of “thermodynamic” equilibrium states. The local energy, probability acceleration, and force concepts are related to the state phase equalization and production. It is stressed that, contrary to the sourceless classical IT measures, the resultant descriptors exhibit finite local productions due to their nonclassical contributions.

## 2. Local Energy and Phase Equalization

Consider, for simplicity reasons, the quantum state |*ψ*(*t*)〉 of a single electron at time *t*, and the associated (complex) wavefunction in position representation,

*ψ*(***r***, *t*) = 〈***r***|*ψ*(*t*)〉 = *R*(***r***, *t*) exp[i*φ*(***r***, *t*)], (1)

defined by its modulus *R*(***r***, *t*) and phase *φ*(***r***, *t*) ≥ 0 parts. The state logarithm then additively separates these two independent components:

2ln*ψ*(***r***, *t*) = 2ln*R*(***r***, *t*) + 2i*φ*(***r***, *t*) = ln*p*(***r***, *t*) + 2i*φ*(***r***, *t*), (2)

where *p*(***r***, *t*) = *R*(***r***, *t*)^2^ denotes the particle spatial probability density. Its real part determines the logarithm of the state classical (probability) component, while the imaginary part accounts for the nonclassical (phase) distribution:

Re[2ln *ψ*(***r***, *t*)] = 2ln*R*(***r***, *t*) = ln*p*(***r***, *t*) and Im[ln*ψ*(***r***, *t*)] = *φ*(***r***, *t*). (3)

The electron is moving in the external potential *v*(***r***), due to the fixed positions of the system constituent nuclei. In this Born–Oppenheimer (BO) approximation the (Hermitian) electronic Hamiltonian

H(***r***) = −[*ħ*^2^/(2*m*)]Δ + *v*(***r***) ≡ T(***r***) + *v*(***r***) (4)

determines the quantum dynamics of this molecular state, in accordance with the time-dependent SE.

i*ħ* [*∂ψ*(***r***, *t*)/∂*t*] = H(***r***) *ψ*(***r***, *t*). (5)

This fundamental equation and its complex conjugate ultimately imply the associated dynamic equations for the wavefunction components or temporal evolutions of the associated physical distributions of the spatial probability and current densities (see the next section).

Consider the *stationary* state corresponding to the sharply specified energy *E_st._*,

*ψ_st._*(***r***, *t*) = *R_st._*(***r***) exp[−i(*E_st._*/*ħ*)*t*] = *R_st._*(***r***) exp(−i*ω_st._t*) (6)

where *φ_st._*(***r***, *t*) = −*ω_st._t* ≡ *φ_st._*(*t*). In this state the probability distribution is *time*-independent,

*p_st._*(***r***, *t*) = |*ψ_st._*(***r***, *t*)|^2^ = *R_st._*(***r***)^2^ ≡ *p_st._*(***r***), (7)

and the probability current exactly vanishes:

***j****_st._*(***r***, *t*) = (*ħ*/2*m*i) {*ψ_st._*(***r***, *t*)^*^ ∇*ψ_st._*(***r***, *t*) − *ψ_st._*(***r***, *t*) ∇*ψ_st._*(***r***, *t*)^*^} = (*ħ*/*m*) *p_st._*(***r***) ∇*φ_st._*(*t*) = 0. (8)

These eigenstates of electronic Hamiltonian,

H(***r***) *ψ_st._*(***r***, *t*) = *E_st._ ψ_st._*(***r***, *t*) or H(***r***) *R_st._*(***r***) = *E_st._ R_st._*(***r***) (9)

correspond to the spatially equalized local energy

*E*(***r***, *t*) ≡ *ψ*(***r***, *t*)^−1^ H(***r***) *ψ*(***r***, *t*), (10)

*E_st._*(***r***) ≡ *R_st._*(***r***)^−1^ H(***r***)*R_st._*(***r***) = *E_st._.* (11)

This equalization principle can be also interpreted as the related equalization rule for the state spatial phase. Indeed, introducing the local wave-number/phase concepts,

*ω*(***r***, *t*) ≡ *E*(***r***, *t*)/*ħ* and *φ*(***r***, *t*) = −*ω*(***r***, *t*) *t*, (12)

directly implies their spatial equalization in the stationary electronic state:

*ω_st._*(***r***, *t*) = *E_st._*/*ħ* = *ω_st._* = *const.* and

*φ_st._*(***r***, *t*) = −(*E_st._*/*ħ*)*t* = −*ω_st._t* = *φ_st._*(*t*). (13)

The *stationary* equilibrium in QM is thus marked by the local phase equalization throughout the whole physical space. It should be realized that due to the complex nature of wavefunctions, the local energy of Equation (10) is also complex in character: *E*(***r***, *t*) ≠ *E*(***r***, *t*)^*^. This further implies the complex concepts of the local phase or wave-number,

*ω*(***r***, *t*) = *c*(***r***, *t*) + i *b*(***r***, *t*), *c*(***r***, *t*) = Re[*ω*(***r***, *t*)] = [*ω*(***r***, *t*) + *ω*(***r***, *t*)^*^]/2, *b*(***r***, *t*) = Im[*ω*(***r***, *t*)] = [*ω*(***r***, *t*) − *ω*(***r***, *t*)^*^]/(2i), (14)

which determines dynamic equations for the additive components of the state wavefunction of Equations (2) and (3). Rewriting SE in terms of complex wave-number components gives:

*∂*ln*ψ*(***r***, *t*)/∂*t* = *ψ*(***r***, *t*)^−1^ [*∂ψ*(***r***, *t*)/∂*t*] = *∂*ln*R*(***r***, *t*)/∂*t* + i *∂φ*(***r***, *t*)/∂*t* = −i*ω*(***r***, *t*) = −i*c*(***r***, *t*) + *b*(***r***, *t*). (15)

The real terms in this complex equation determine the *modulus* dynamics,

*∂*ln*R*(***r***, *t*)/∂*t = b*(***r***, *t*), (16)

while its imaginary terms determine the time evolution of the wavefunction phase:

*∂φ*(***r***, *t*)/∂*t* = −*c*(***r***, *t*). (17)

For more SE identification of these wave-number components, the reader is referred to Equations (65) and (66) in Section 5.

To summarize, the (complex) local energy generates a transparent description of the time evolution of wave-function components: its real contribution shapes the *phase* dynamics, while the *modulus* dynamics is governed by the imaginary components of *E*(***r***, *t*) or *ω*(***r***, *t*). In QM the spatial equalization of these wave-number or local-phase concepts marks the stationary state corresponding to the sharply specified energy, purely time-dependent phase, and time-independent probability distribution. We argue in Section 7 and Section 8 that these equilibrium states may still exhibit finite “hidden” flows of electrons, along probability contours, which can be associated with the local “horizontal” phase defining the *phase*-transformed, “thermodynamic” states.

## 3. Origins of Information Content in Electronic States

The independent (real) parts of the complex electronic wavefunction of an electron in Equation (1) ultimately define the state physical descriptors of the spatial probability density *p*(***r***, *t*) = *R*(***r***, *t*)^2^ and its current

***j***(***r***, *t*) = (*ħ*/*m*) *p*(***r***, *t*) ∇*φ*(***r***, *t*) ≡ *p*(***r***, *t*) ***V***(***r***, *t*). (18)

The effective probability velocity introduced in the preceding equation measures a density of the current-per-particle,

***V***(***r***, *t*) ≡ ***P***(***r***, *t*)/*m* = (*ħ*/*m*) ∇*φ*(***r***, *t*) ≡ ***j***(***r***, *t*)/*p*(***r***, *t*), (19)

and reflects the local convection momentum ***P***(***r***, *t*) ≡ *ħ* ***k***(***r***, *t*), with ***k***(***r***, *t*) = ∇*φ*(***r***, *t*) standing for its wave-vector factor.

The real and imaginary components of Equation (3), in the wavefunction logarithm of Equation (2), determine the independent probability and velocity densities, respectively. They account for the “static” and “dynamic” (convection) aspects of the state probability distribution, which we call the molecular structures of “being” and “becoming”. Both these organization levels ultimately contribute to the overall entropy or gradient-information contents in quantum electronic states and their thermodynamic mixtures [2,10,11,12,13,14].

The probability IT functionals *S*[*p*] and *I*[*p*], due to the logarithm of the state probability density of Equation (2), constitute the classical IT concepts of Shannon’s global entropy [34,35],

*S*[*p*] = −∫*p*(***r***, *t*) ln*p*(***r***, *t*) *d**r***, (20)

and Fisher’s information functional for locality events [36,37]:

*I*[*p*] = ∫*p*(***r***, *t*) [∇ln*p*(***r***, *t*)]^2^*d**r*** = ∫*p*(***r***, *t*)^−1^ [∇*p*(***r***, *t*)]^2^*d**r***. (21)

In the associated resultant measures [2,10,11,12,13,14] these probability functionals are supplemented by the average nonclassical contributions *S*[*φ*] and *I*[*φ*] = *I*[***j***], due to the state phase or its gradient generating the probability velocity:

*S*[*ψ*] = *S*[*p*] − 2∫*p*(***r***, *t*) *φ*(***r***, *t*) *d**r*** ≡ *S*[*p*] − 2〈*φ*〉*_ψ_* = *S*[*p*] + *S*[*φ*] = *S*[*p*, *φ*], and

*I*[*ψ*] = *I*[*p*] + 4∫*p*(***r***, *t*) [∇*φ*(***r***, *t*)]^2^*d**r*** = *I*[*p*] + *I*[*φ*] = *I*[*p*, *φ*] = *I*[*p*] + (2*m*/*ħ*)^2^ ∫*p*(***r***, *t*)^−^^1^***j***(***r***, *t*)^2^*d**r*** = *I*[*p*] + *I*[***j***] = *I*[*p*, ***j***]. (22)

We also introduce the combined measure of the gradient-entropy,

*M*[*ψ*] = *M*[*p*] + *M*[*φ*] ≡ *I*[*p*] − *I*[*φ*]. (23)

The nonclassical entropy terms *S*[*φ*] and *M*[*φ*] ≡ −*I*[*φ*] = −*I*[***j***] are negative since the current pattern introduces an extra dynamic “order” into the system electronic “organization”, compared to the corresponding classical descriptors *S*[*p*] and *M*[*p*] = *I*[*p*], thus decreasing the state overall “uncertainty” content. These generalized descriptors of the resultant uncertainty (entropy) content *S*[*ψ*] in the quantum state *ψ*, or of its overall (gradient) information *I*[*ψ*] [2,10,11,12,13,14], have been used to describe the phase equilibria in the substrate subsystems and to monitor electronic reconstructions in chemical reactions [3,4,5,13,14,38,39,40].

To summarize, in the resultant IT descriptors of the pure quantum state *ψ*, the classical probability functionals, of Shannon’s global entropy or Fisher’s intrinsic accuracy for locality events, are supplemented by the corresponding nonclassical complements *S*[*φ*] or *I*[*φ*] = *I*[***j***], respectively, due to the wavefunction phase or the electronic current it generates. In the overall (“scalar”) entropy [2,10], the (positive) classical descriptor is combined with the (negative) average phase contribution,

*S*[*ψ*] *=* −∫*p*(***r***, *t*)[ln *p*(***r***, *t*) + 2*φ*(***r***, *t*)] *d**r*** ≡ ∫*p*(***r***, *t*) *S*(***r***, *t*) *d**r***. (24)

while the complex (“vector”) entropy [2,12] represents the expectation value of the state (non-Hermitian) entropy operator **S** = −2ln*ψ*:

***S***[*ψ*] = 〈*ψ*|**S**|*ψ*〉 *=* − ∫*p*(***r***, *t*)[ln *p*(***r***, *t*) + 2i*φ*(***r***, *t*)] *d**r*** ≡ ∫*p*(***r***, *t*) ***S***(***r***, *t*) *d**r*** ≡ *S*[*p*] + i *S*[*φ*] = ***S***[*p*, *φ*]. (25)

Therefore, the negative nonclassical entropy effectively lowers the state classical uncertainty measure *S*[*p*]. Indeed, the presence of finite currents implies more state spatial “order”, i.e., less electronic “disorder”. The resultant measure of the state average gradient information [2,10,11,12,13,14,15],

*I*[*ψ*] = 4〈∇*ψ*|∇*ψ*〉 = −4〈*ψ*|Δ|*ψ*〉 = (8*m*/*ħ*^2^)〈*ψ*|T|*ψ*〉 ≡ *κT*[*ψ*] = ∫*p*(***r***, *t*){[∇ln*p*(***r***, *t*)]^2^ + 4[∇*φ*(***r***, *t*)]^2^} *d**r*** ≡ ∫*p*(***r***, *t*) *I*(***r***, *t*) *d**r***, (26)

then reflects the (dimensionless) kinetic energy of electrons: *T*[*ψ*] = 〈*ψ*|T|*ψ*〉 = *κ*^−1^ *I*[*ψ*].

**Figure 1:**
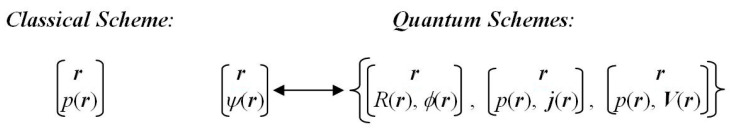
Classical (probability) and quantum (wavefunction) information schemes in molecular QM. The quantum mapping {***r*** → *ψ*(***r***)} implies both the classical {***r***→*p*(***r***)} and nonclassical attributions {***r***→[*φ*(***r***), ***j***(***r***) or ***V***(***r***)]}.

In both the classical IT and in position representation of QM the admissible locations {***r***} of an electron exhaust the whole physical space and constitute the complete set of elementary particle-position events. The associated infinite and continuous *probability scheme* of the classical mapping {***r***→*p*(***r***)} in Figure 1 thus describes a state of the position *indeterminacy* (uncertainty). It is best reflected by Shannon’s global entropy *S*[*p*], measuring a “spread” (width) of the probability distribution, since we know only the probabilities *p*(***r***) = |*ψ*(***r***)|^2^ of possible definite outcomes of the underlying localization experiment in the pure quantum state *ψ*. Another suitable classical probe of the average information content in *p*(***r***) is provided by Fisher’s probability functional *I*[*p*]. This gradient measure of the position *determinacy* reflects the “compactness” (height) of the probability distribution, thus complementing the Shannon global descriptor.

The information given us by carrying out the given experiment consists of removing the uncertainty existing before the experiment [32]. If we carry out the particle-localization probe we obtain some information, since its outcome means that we then know exactly, which position has actually been detected. This implies that, after repeated trials performed for the specified quantum state, the initial uncertainty contained in the position *probability scheme* has been completely eliminated. The average information gained by such tests thus amounts to the removed position uncertainty. The larger the uncertainty in *p*(***r***), the larger the amount of information obtained when we eventually find out which electron position has actually been detected after the experiment. In other words, the amount of information given us by the realization of the classical, probability scheme alone equals the global entropy in the classical probability scheme of Figure 1 [31,32].

In QM, however, one deals with the *wavefunction scheme* {***r*** → *ψ*(***r***)} of Figure 1, in which the classical probability map {***r*** → *p*(***r***)} constitutes only a part of the overall (complex) mapping. In fact, the wavefunction mapping implies a simultaneous ascription to the parametrically specified electron position of the local modulus (static) and phase/current (dynamic) arguments of the state wavefunction, or the related local probability and probability velocity descriptors. This two-level scheme in QM ultimately calls for the *resultant* measures of the entropy/information content in quantum states, combining classical (probability) and nonclassical (phase/current/velocity) contributions. The difference between the resultant and classical information contents can be best compared to that between the (phase-dependent) hologram and (phase-independent) ordinary photograph.

The resultant IT measures are in principle experimentally accessible, since the local probability velocity in physical space, defined by the velocity of probability current, is uniquely specified in QM. In other words, all static and dynamic arguments of the resultant IT descriptors are all sharply specified by the corresponding expectation values of the associated observables. However, the localization experiment alone cannot remove all the uncertainty contained in a general electronic state, which exhibits a nonvanishing local phase component *φ*(***r***, *t*) and hence gives rise to a finite current density ***j***(***r***, *t*). This probability flux vanishes only in the stationary state of Equation (6), for the purely time-dependent stationary phase *φ_st._*(*t*): ***j****_st._*(***r***, *t*) = ***V****_st._*(***r***, *t*) = ***0***. For such states an experimental determination of electronic position removes completely all the uncertainty contained in the spatial wavefunction *R_st._*(***r***) and the probability distribution *p_st._*(***r***) = *R_st._*(***r***)^2^. Indeed, the quantum scheme of Figure 1 then reduces to the classical mapping alone.

Since the current operator **j**(***r***) includes the momentum operator of an electron, **P**(***r***) = −i*ħ*∇,

**j**(***r***) = (2*m*)^−1^[**P**p(***r***) + p(***r***)**P**], p(***r***) = |***r***〉〈***r***|, {**r** |***r***’〉 = ***r***’|***r***’〉}, (27)

which does not commute with the position operator **r**(***r***) = ***r***,

**Pr** − **rP** ≡ [**P**, **r**] = −i*ħ*, (28)

the incompatible observables **r** and **j**(***r***) do not have common eigenstates. In other words, these quantities cannot be *simultaneously* defined sharply, in accordance with Heisenberg’s uncertainty principle of QM. Therefore, the position dispersion *σ**_r_*** cannot be simultaneously eliminated with the current dispersion *σ**_j_*** in a single type of experiment, e.g., that of the particle localization. Indeed, a removal of *σ**_j_*** ultimately calls for an additional momentum experimental setup, which is incompatible with that required for determining the electronic position. Only the repeated, *separate* localization and momentum experiments, performed on molecular systems in the same quantum state, can fully eliminate the position and current uncertainties contained in a general electronic state. Neertheless, both the particle position ***r*** and the local convection velocity ***V***(***r***) of the probability distribution are precisely defined as expectation values of the associated Hermitian operators. Therefore, their resultant IT functionals are all uniquely specified, with their densities exhibiting vanishing spatial dispersions.

The nonclassical *uncertainty S*[*φ*], proportional to the state average phase 〈*φ*〉*_ψ_*, effectively lowers the *information* received from the localization-only experiment. The removable uncertainty in *ψ*(***r***) is then less than its classical content *S*[*ρ*] or *M*[*ρ*] = *I*[*ρ*]. In other words, the nonvanishing current pattern introduces an extra (dynamic) *determinacy* in the system electronic structure, which diminishes its resultant uncertainty (*indeterminacy*) descriptors.

The *phase* equilibria corresponding to *phase*-transformed quantum states,

*ψ_eq._*(***r***) = *ψ*(***r***) exp{i*φ_eq._*[*p*, ***r***]}, (29)

have been explored elsewhere [2,10,11,12,13,14]. The optimum local (“thermodynamic”) phase component *φ_eq._*[*p*, ***r***] ≡ *φ_eq._*(***r***) for the specified probability density *p*(***r***) *= p_st._*(***r***) in the stationary state *ψ* = *ψ_st._* of Equation (6) marks the exact cancellation of the state classical (*S*[*p*]) and nonclassical (*S*[*φ_eq._*]) entropy contributions:

*S*[*ψ_eq._*] = *S*[*p*] + *S*[*φ_eq._*] = − ∫*p*(***r***) [ln*p*(***r***) + 2*φ_eq._*(***r***)]*d**r*** = 0. (30)

We argue in the next section that this exact reduction of the “internal” (resultant) entropy content in the equilibrium “thermodynamic” state is essential for the consistency between the von Neumann thermodynamic entropy [33] and the overall IT entropy in the *grand* ensemble. 

The above condition determines the equilibrium (“thermodynamic”, horizontal) local phase for the conserved (stationary) probability distribution,

*p_eq._*(***r***) = |*ψ_eq._*(***r***)|^2^ = |*ψ*(***r***)|^2^ = *p*(***r***), (31)

proportional to the negative logarithm of probability density:

*φ_eq._*[*p*, ***r***] = − ½ ln*p*(***r***) ≥ 0. (32)

The same prediction follows from the condition of the vanishing gradient measure of the resultant entropy content in *ψ_eq._*:

*M*[*ψ_eq._*] = *M*[*p*] + *M*[*φ_eq._*] = *I*[*p*] − *I*[*φ_eq._*] = ∫*p*(***r***) {[∇ln*p*(***r***)]^2^ − 4[∇*φ_eq._*(***r***)]^2^} *d**r*** = 0. (33)

Indeed, solving this equation for *φ_eq._* ≥ 0 (phase convention) gives:

[∇ln*p*(***r***)]^2^ − 4[∇*φ_eq._*(***r***)]^2^ = [∇ln*p*(***r***) − 2∇*φ_eq._*(***r***)] [∇ln *p*(***r***) + 2∇*φ_eq._* (***r***)] = 0 or

∇ln*p*(***r***) + 2*φ_eq._*(***r***) = 0 ⇒ *φ_eq._*(***r***) = − ½ ln*p*(***r***). (34)

We can also observe that writing the average functionals for resultant entropy measures as expectations of the corresponding (multiplicative) operators,

*S*[*ψ*] = −∫*p*(***r***) [ln*p*(***r***) + 2*φ*(***r***)] *d**r*** ≡ −〈*ψ*|ln*p* + 2*φ*|*ψ*〉

and

*M*[*ψ*] = ∫*p*(***r***) {[∇ln*p*(***r***)]^2^ − 4[∇*φ_eq._*(***r***)]^2^} *d**r*** = 〈*ψ*|(∇ln*p*)^2^ − 4(∇*φ*)^2^|*ψ*〉, (35)

makes it possible to formally interpret the equilibrium phase of Equations (32) and (34) as the optimum solution defined by the extrema of these *wavefunction* functionals:

{*δS*[*ψ*]/*δψ*(***r***)* = 0 or *δM*[*ψ*]/*δψ*(***r***)* = 0} ⇒ *φ_eq._*(***r***) = −½ ln*p*(***r***). (36)

## 4. Equilibrium States and Thermodynamic Entropy

Consider now the mixed quantum state in the *grand* ensemble, the statistical mixture of molecular stationary states {|Ψ*_j_^i^*〉 ≡ |Ψ*_j_*(*N_i_*)〉} for different numbers of electrons {*N_i_*}, defined by the corresponding density operator,

D = ∑*_i_*∑*_j_* |Ψ*_j_^i^*〉*P_j_^i^*〈Ψ*_j_^i^*| ≡ ∑*_i_*∑*_j_P_j_^i^* O*_j_^i^*, ∑*_i_*∑*_j_* O*_j_^i^* = 1, ∑*_i_*∑*_j_P_j_^i^* ≡ ∑*_i_P^i^* = 1, (37)

where, O*_j_^i^* = |Ψ*_j_^i^*〉〈Ψ*_j_^i^*| stands for the state projector. The average entropy or information—say, the resultant IT quantity *G* represented by the associated operator G, possibly state-dependent, G = G[Ψ*_j_^i^*] ≡ G*_j_^i^*, is given by the weighted average of the property state-expectations {*G_j_^i^* = 〈Ψ*_j_^i^*|G|Ψ*_j_^i^*〉}:

〈*G*〉*_ens._* = tr(DG) = ∑*_i_*∑*_j_P_j_^i^* 〈Ψ*_j_^i^*|G|Ψ*_j_^i^*〉 ≡ ∑*_i_*∑*_j_P_j_^i^ G_j_^i^* ≡ 𝓖(D). (38)

For example, the ensemble entropy of von Neumann [33],

〈*S*〉*_ens._* = −*k*_B_ ∑*_i_*∑*_j_P_j_^i^* ln*P_j_^i^* ≡ 𝓢(D), (39)

corresponds to the state entropy operator S*_j_^i^* = *S_j_^i^* O*_j_^i^* and the expectation value of entropy in state Ψ*_j_^i^*

*S_j_^i^ =* 〈Ψ*_j_^i^*|S*_j_^i^*|Ψ*_j_^i^*〉 = −*k*_B_ ln*P_j_^i^*. (40)

This average value depends solely on the state *external* probability *P_j_^i^* in the mixture, shaped by thermodynamic conditions, and is devoid of any local (*internal*) content of the constituent wavefunction distributions.

One would expect a similar feature in the overall IT description of molecular ensembles. In the pure quantum state |Ψ*_j_^i^*〉 the probability of finding an electron at the specified location ***r*** is given by the state internal distribution,

*p_j_^i^*(***r***) = *ρ_j_^i^*(***r***)/*N_i_* ≡ 〈Ψ*_j_^i^*|p(***r***)|Ψ*_j_^i^*〉, (41)

the shape factor of the associated electron density *ρ_j_^i^*(***r***). In thermodynamic ensemble it is given by the weighted average over such internal state densities {*p_j_^i^*(***r***)}, with the state (external) probability weights {*P_j_^i^*}:

〈*p*(***r***)〉*_ens._* = tr(Dp) = ∑*_i_*∑*_j_P_j_^i^ p_j_^i^*(***r***) ≡ ∑*_i_*∑*_j_ P*(Ψ*_j_^i^*, ***r***). (42)

The probability product *P*(Ψ*_j_^i^*, ***r***) represents the normalized *joint* probability of finding in state Ψ*_j_^i^* an electron at ***r***, with both its factors thus acquiring the status of *conditional* probabilities:

*P_j_^i^* ≡ *P*(Ψ*_j_^i^*|***r***) = *P*(Ψ*_j_^i^*, ***r***)/*p_j_^i^*(***r***), ∑*_i_*∑*_j_ P*(Ψ*_j_^i^*|***r***) = 1; *p_j_^i^*(***r***) ≡ *P*(***r***|Ψ*_j_^i^*) = *P*(Ψ*_j_^i^*, ***r***)/*P_j_^i^*, ∫*P*(***r***|Ψ*_j_^i^*) *d**r*** = 1. (43)

The Shannon entropy in the ensemble joint distribution then separates into the “external” entropy *S*[{*P_j_^i^*}] of von Neumann and the weighted average of “internal” state contributions

{*S*[*p_j_^i^*] = − ∫*p_j_^i^*(***r***) ln*p_j_^i^*(***r***) *d**r***}, (44)

*S*[{*P*(Ψ*_j_^i^*, ***r***)}] = − ∑*_i_*∑*_j_* ∫{*P*(Ψ*_j_^i^*, ***r***) ln*P*(Ψ*_j_^i^*, ***r***)} *d**r*** = − ∑*_i_*∑*_j_ P_j_^i^* ln*P_j_^i^* − ∑*_i_*∑*_j_ P_j_^i^* ∫*p_j_^i^*(***r***) ln*p_j_^i^*(***r***) *d**r*** = *S*[{*P_j_^i^*}] + ∑*_i_*∑*_j_ P_j_^i^ S*[*p_j_^i^*]. (45)

For a consistent IT description of the equilibrium *mixed* states of the open reactive complexes and their substrate subsystems, it would be desirable that in each *phase*-transformed *pure* state,

Ψ*_eq._*[*p_j_^i^*] = Ψ*_j_^i^*exp{i*φ_eq._*[*p_j_^i^*]}, (46)

defined by its local (horizontal) phase *φ_eq._*[*p_j_^i^*, ***r***] ≡ *φ*^(*h*)^(***r***) (see also Section 7 and Section 8), equilibrium for the specified state probability density *p_j_^i^*(***r***), the second (internal) contribution of Equation (45), exactly vanishes. This is indeed the case when the internal entropy of each equilibrium state is exactly zero:

*S*[Ψ*_eq._*[*p_j_^i^*]] = *S*[*p_j_^i^*] − 2 ∫*p_j_^i^*(***r***) *φ_eq._*[*p_j_^i^*, ***r***] *d**r*** ≡ 0. (47)

In statistical mixtures of the equilibrium stationary states the only source of uncertainty is then generated by von Neumann’s ensemble entropy, determined by the “external” probabilities alone. This consistency requirement thus identifies the state equilibrium phase of Equation (32) [2,10,11,12,13,14]:

*φ_eq._*[*p_j_^i^*, ***r***] = − ½ ln*p_j_^i^*(***r***) ≥ 0. (48)

In such “horizontally” *phase*-transformed states the thermodynamic and resultant equilibrium entropies are thus consistent with one another:

〈*S*〉*_ens._* = *k*_B_*S*[{*P*(Ψ*_j_^i^*, ***r***)}] = *k*_B_*S*[{*P_j_^i^*}]. (49)

To summarize, the equilibrium “thermodynamic” (horizontal) phase is proportional to the local probability logarithm. This is very much in spirit of density-functional theory (DFT) [41,42,43,44,45,46]: the equilibrium stationary state is the unique functional of the system electron distribution *ρ_j_^i^*(***r***) = *N_i_ p_j_^i^*(***r***), Ψ*_j_^i^*^,*eq.*^ = Ψ*_eq._*[*ρ_j_^i^*], since both Ψ*_j_^i^* = Ψ*_j_^i^*[*ρ_j_^i^*], by the first Hohenberg–Kohn (HK) [41] theorem, and the equilibrium “thermodynamic” phase *φ_eq_*_._ = *φ_eq._*[*p_j_^i^*].

Therefore, when the state “thermodynamic” phase satisfies the “equilibrium” criterion of Equation (30), the introduction of the *phase*-transformed states for conserved (stationary) probability distribution generates the mutual consistency between the external (ensemble) and internal (resultant) entropy descriptors. It implies that for the single stationary state the resultant global and gradient uncertainty descriptors of the specified wavefunction vanish in equilibrium, as indeed does von Neumann’s [33] entropy of the pure quantum state. In such states, the internal nonclassical (phase/current) contribution exactly cancels out the classical (probability) term. The equilibrium-phase condition of the state vanishing “internal” (resultant) IT descriptor then consistently predicts the equilibrium (horizontal) phase being related to the negative logarithm of the stationary probability distribution [2,10,11,12,13,14]:

{*M*[*p_st_*_._, *φ*^(*h*)^] = 0 or *S*[*p_st_*_._, *φ*^(*h*)^] = 0} ⇒ *φ_opt._*^(*h*)^(***r***) = − ½ ln*p_st_*_._(***r***) ≡ *φ_eq._*(*p_st_*_._). (50)

## 5. Continuity Relations

It is of crucial importance for continuity laws of QM to distinguish between the reference frame moving with the particle (Lagrangian frame) and the reference frame fixed to the prescribed coordinate system (Eulerian frame). The total derivative *d*/*dt* is the time change appearing to an observer who moves with the probability flux, while the partial derivative ∂/∂*t* is the local time rate of change observed from a fixed point in the Eulerian reference. These derivatives are related to each other by the chain-rule transformation,

*d*/*dt* = ∂/∂*t* + ***V***(***r***, *t*)⋅∇, (51)

where the velocity-dependent part ***V***(***r***, *t*) ⋅∇ generates the probability “convection” term.

In Schrödinger’s dynamical picture the state vector |*ψ*(*t*)〉 introduces an *explicit* time dependence of the system wavefunction, while the dynamics of the basis vector |***r***(*t*)〉 of the position representation is the source of an additional, *implicit* time dependence of the electronic wavefunction *ψ*(***r***, *t*) = *ψ*[***r***(*t*), *t*], due to the moving reference (monitoring) point. This separation applies to wavefunctions, their components, and expectation values of physical observables. In Table 1 we summarize the dynamic equations for the wavefunction modulus and phase components together with the continuity relations for the state probability, current, and information densities, which directly follow from the wavefunction dynamics of SE.

It directly follows from the SE that the probability field is sourceless:

*∂p*(***r***, *t*)/∂*t* = 2*R*(***r***, *t*) [*∂R*(***r***, *t*)/∂*t*] = −∇⋅ ***j***(***r***, *t*) = − ***V***(***r***, *t*)⋅∇*p*(***r***, *t*) or

*σ_p_*(***r***, *t*) ≡ *dp*(***r***, *t*)/*dt* = *∂p*(***r***, *t*)/∂*t* + ∇⋅***j***(***r***, *t*) = *∂ρ*(***r***, *t*)/∂*t* + ∇*p*(***r***, *t*)⋅***V***(***r***, *t*) = 0. (52)

Indeed, separating the explicit and implicit time dependencies in probability density *p*(***r***, *t*) = *p*[***r***(*t*), *t*] gives:

*σ_p_*(***r***, *t*) = ∂*p*[***r***(*t*), *t*]/∂*t* + (*d**r***/*dt*)⋅∂*p*(***r***, *t*)/∂***r*** = ∂*p*(***r***, *t*)/∂*t* + ***V***(***r***, *t*)⋅∇*p*(***r***, *t*) = ∂*p*(***r***, *t*)/∂*t* + ∇⋅***j***(***r***, *t*). (53)

Above, the total *time* derivative *dp*(***r***, *t*)/*dt* determines the vanishing local probability “source”: *σ_p_*(***r***, *t*) = 0. It measures the time rate of change in an infinitesimal volume element of probability fluid *moving* with probability velocity ***V***(***r***, *t*) = *d**r***(*t*)/*dt*, while the partial derivative ∂*p*[***r****(t*), *t*]/∂*t* refers to a volume element around the *fixed* point in space. The divergence of probability flux in the preceding equation,

∇⋅***j***(***r***, *t*) = ∇*p*(***r***, *t*)⋅***V***(***r***, *t*) + *p*(***r***, *t*)∇⋅***V***(***r***, *t*) = ∇*p*(***r***, *t*)⋅***V***(***r***, *t*), (54)

thus implies the vanishing divergence of the velocity field ***V***(***r***, *t*), related to the phase Laplacian ∇^2^*φ*(***r***, *t*) = Δ*φ*(***r***, *t*):

∇⋅***V***(***r***, *t*) = (*ħ*/*m*) Δ*φ*(***r***, *t*) = 0 or Δ*φ*(***r***, *t*) = 0. (55)

As in fluid dynamics, in these transport equations the operators (***V***⋅∇) and ∇^2^ = Δ represent the “convection” and “diffusion”, respectively. Thus, in Equation (52), the local evolution of the particle probability is governed by the density “convection”, while the preceding equation implies the vanishing “diffusion” of the phase distribution.

In Table 1 we summarize local continuity equations for the wavefunction components, the state physical descriptors, and information densities. For example, it follows from the table that the resultant gradient information exhibits a nonvanishing net production *σ_I_*(*t*) due to a finite phase source *σ_φ_*(***r***, *t*). The classical contribution to *σ_I_*(*t*) identically vanishes due to the probability continuity of Equation (52). These relations directly follow from the molecular SE and identify the relevant local sources of the distributions of interest. 

As an example, consider continuities of the wavefunction components. When expressed in terms of the state modulus and phase parts the SE reads:

i*ħ* [*∂ψ*(***r***, *t*)/∂*t*] = i*ħ* {[*∂R*(***r***, *t*)/∂*t*] + i *R*(***r***, *t*) [*∂φ*(***r***, *t*)/∂*t*]} exp[i *φ*(***r***, *t*)] = H(***r***) *ψ*(***r***, *t*) = [−*ħ*^2^(2*m*)^−1^{Δ*R*(***r***, *t*) + 2i∇*R*(***r***, *t*) ⋅∇*φ*(***r***, *t*) − *R*(***r***, *t*) [∇*φ*(***r***, *t*)]^2^} + *v*(***r***) *R*(***r***, *t*)] exp[i*φ*(***r***, *t*)], (56)

where we have used Equation (55). Dividing both sides by *ħR*(***r***, *t*) and multiplying by exp[−i*φ*(***r***, *t*)] gives the following (complex) dynamic relation linking the wavefunction components:

i [*∂*ln*R*(***r***, *t*)/∂*t*] − *∂φ*(***r***, *t*)/∂*t* = −[*ħ*/(2*m*)]{*R*(***r***, *t*)^−1^Δ*R*(***r***, *t*) + 2i[∇ln*R*(***r***, *t*)]⋅∇*φ*(***r***, *t*) − [∇*φ*(***r***, *t*)]^2^} + *v*(***r***)/*ħ*. (57)

Comparing its *imaginary* parts generates the time evolution of the modulus part of electronic state,

*∂*ln*R*(***r***, *t*)/∂*t* = − (*ħ*/*m*) ∇*φ*(***r***, *t*) ⋅∇ln*R*(***r***, *t*) = − ***V***(***r***, *t*) ⋅∇ln*R*(***r***, *t*), (58)

which can be directly transformed into the probability continuity equation

*∂p*(***r***, *t*)/∂*t* = −∇⋅***j***(***r***, *t*) or *σ_p_*(***r***, *t*) = *dp*(***r***, *t*)/*dt* = 0. (59)

Equating the *real* parts of Equation (57) similarly determines the phase dynamics

*∂φ*(***r***, *t*)/∂*t* = [*ħ*/(2*m*)] {*R*(***r***, *t*)^−1^Δ*R*(***r***, *t*) − [∇*φ*(***r***, *t*)]^2^} − *v*(***r***)/*ħ*. (60)

The preceding equation ultimately determines the production term *σ_φ_*(***r***, *t*) = *dφ*(***r***, *t*)/*dt* in the *phase*-continuity relation

*∂φ*(***r***, *t*)/∂*t* = −∇⋅***J***(***r***, *t*) + *σ_φ_*(***r***, *t*), (61)

since the effective velocity ***V***(***r***, *t*) of the *probability* current ***j***(***r***, *t*) = *p*(***r***, *t*) ***V***(***r***, *t*) also determines the *phase* flux and its divergence, the convection term in the continuity Equation (61):

***J***(***r***, *t*) = *φ*(***r***, *t*) ***V***(***r***, *t*) and ∇⋅***J***(***r***, *t*) = ***V***(***r***, *t*) ⋅∇*φ*(***r***, *t*) = (*ħ*/*m*) [∇*φ*(***r***, *t*)]^2^. (62)

This complementary flow descriptor ultimately identifies the finite phase production

*σ_φ_*(***r***, *t*) ≡ *dφ*(***r***, *t*)/*dt* = ∂*φ*(***r***, *t*)/∂*t* + ***V***(***r***, *t*) ⋅ ∇*φ*(***r***, *t*) ≠ 0. (63)

Finally, using Equation (60) gives the following expression for the *phase* source:

*σ_φ_*(***r***, *t*) = [*ħ*/(2*m*)]{*R*(***r***, *t*)^−1^Δ*R*(***r***, *t*) + [∇*φ*(***r***, *t*)]^2^} − *v*(***r***)/*ħ*. (64)

This production of the local phase is seen to group the probability-diffusion and phase-convection terms supplemented by the external potential contribution.

The component SE (57) also allows one to identify the wave-number distributions introduced in Equations (14)–(17):

*c*(***r***, *t*) = −*∂φ*(***r***, *t*)/∂*t* = −[*ħ*/(2*m*)] {*R*(***r***, *t*)^−1^Δ*R*(***r***, *t*) − [∇*φ*(***r***, *t*)]^2^} + *v*(***r***)/*ħ* = −[*ħ*/(2*m*)] {Δln*R*(***r***, *t*) + [∇ln*R*(***r***, *t*)]^2^ − [∇*φ*(***r***, *t*)]^2^} + *v*(***r***)/*ħ* (65)

and

*b*(***r***, *t*) = *∂*ln*R*(***r***, *t*)/∂*t* = − (*ħ*/*m*) ∇*φ*(***r***, *t*) ⋅∇ln*R*(***r***, *t*) = − ***V***(***r***, *t*) ⋅∇ln*R*(***r***, *t*). (66)

To summarize, the effective velocity of the probability current also determines the phase flux in molecular states. The source (net production) of the classical *probability* variable of electronic states identically vanishes, while that of their nonclassical *phase* part remains finite. In overall descriptors of the state information or entropy contents they ultimately generate finite production terms. For example, the nonclassical information *I*[*φ*] generates the nonvanishing (integral) source of the average resultant gradient information *I*[*ψ*]:

*σ_I_*(*t*)= *dI*[*φ*]/*dt* ≡ ∫*p*(***r***, *t*)*σ_I_*(***r***, *t*) *d**r*** = (8*m*/*ħ*) ∫ ***j***(***r***, *t*) ⋅∇*σ_φ_*(***r***, *t*) *d**r***. (67)

Its density-per-electron *σ_I_*(***r***, *t*) is determined by a product of the local probability “flux” ***j***(***r***, *t*) and “affinity“ factor proportional to the gradient of the phase source. It also follows from this local information source in Table 1, that it is determined by the “convection” of the phase source *σ_φ_*(***r***, *t*):

*σ_I_*(***r***, *t*) = (8*m*/*ħ*) ***V***(***r***, *t*) ⋅∇*σ_φ_*(***r***, *t*). (68)

## 6. Principle of Stationary Resultant Information and Charge-Transfer Descriptors of Open Systems

The equilibrium subsystems in the specified (pure) state of the molecular system as a whole require the *mixed*-state description in terms of *ensemble*-average physical quantities [31,47,48,49,50]. The same applies to the (externally) open microscopic systems in the applied thermodynamic conditions. In reactivity problems the specified temperature *T* of the “heat bath” *𝕭*(*T*) and electronic chemical potential *μ* (or electronegativity *χ* = −*μ*) of the macroscopic “electron reservoir” 𝓡(*μ*) call for the *grand*-ensemble approach [44,51,52]. The equilibrium quantum state is then represented by the statistical mixture of the system *pure* (stationary) states, defined by the externally imposed (equilibrium) state probabilities. Indeed, only the *ensemble*-average value of the overall number of electrons 𝓝 ≡ 〈*N*〉*_ens._* exhibits a continuous (fractional) spectrum of values justifying the *populational derivatives* defining the reactivity criteria [44,51,52]. The externally open molecule M(*v*), identified by its external potential *v*(***r***) due to the system fixed nuclei, then constitutes a part of the *composed* system 𝓜 = [M(*v*)¦𝓡(*μ*)] consisting of the mutually *open* (microscopic) molecular fragment M(*v*) and an external (macroscopic) electron reservoir 𝓡(*μ*). In the theory of chemical reactivity one adopts such populational derivatives of the system ensemble-average energy and its underlying Taylor expansion in predicting reactivity behavior of molecules (*single*-reactant criteria) or bimolecular reactive systems (*two*-reactant criteria in situ) [44,53,54,55,56,57,58,59].

Such 𝓝-derivatives of electronic energy are indeed involved in definitions of several reactivity criteria, e.g., the chemical potential/electronegativity [44,52,53,54,55,56,57,58,59,60,61,62] or hardness/softness [46,56,57,58,59,63] and Fukui function (FF) [44,56,57,58,59,64] descriptors of the reaction complex. In IT treatments one introduces analogous concepts of the populational derivatives of the ensemble average (resultant) gradient information. Since reactivity phenomena involve electron flows between the *mutually* open (polarized) substrates, only in such a generalized, ensemble framework can one precisely define the relevant reactivity criteria, determine the hypothetical states of the promoted subsystems, and eventually predict effects of their chemical coordination. It has been demonstrated that, in such an ensemble approach, the energetic and information principles are exactly equivalent, giving rise to identical predictions of thermodynamic equilibria, charge relaxation, and average descriptors of molecular systems and their fragments [9,10,30,65,66].

The populational derivatives of the average energy and resultant information in reactive systems thus invoke the composite representation 〈M(*v*)〉*_ens._* of the equilibrium state of the molecular system M(*v*) in the grand ensemble. Thermodynamic conditions in the (microscopic) molecular system are thus imposed by the hypothetical (macroscopic) heat bath *𝕭*(*T*) and external electron reservoir 𝓡(*μ*). The mixed state then corresponds to the equilibrium probabilities **P**(*μ*, *T*; *v*) ≡ {*P_j_^i^*(*μ*, *T*; *v*)} of the pure (stationary) states {|Ψ*_j_^i^*〉 ≡ |Ψ*_j_*(*N_i_*)〉}, with |Ψ*_j_^i^*〉 denoting the *j*-th state for *N_i_* (integer) number of electrons, which define the equilibrium density operator of Equation (37):

D(*μ*, *T*; *v*) = ∑*_i_*∑*_j_* |Ψ*_j_^i^*〉 *P_j_^i^*(*μ*, *T*; *v*) 〈Ψ*_j_^i^*|, ∑*_i_*∑*_j_P_j_^i^*(*μ*, *T*; *v*) ≡ ∑*_i_P^i^*(*μ*, *T*; *v*) = 1. (69)

This statistical mixture of molecular states gives rise to the ensemble average values of the system electronic energy and its resultant gradient information. The former is defined by the quantum expectations of electronic Hamiltonians {H*_i_* = H(*N_i_*, *v*)},

〈*E*〉*_ens._* = ∑*_i_*∑*_j_ P_j_^i^*(*μ*, *T*; *v*) 〈Ψ*_j_^i^*| H*_i_*|Ψ*_j_^i^*〉 ≡ ∑*_i_*∑*_j_ P_j_^i^*(*μ*, *T*; *v*) *E_j_^i^* ≡ 𝓔(*μ*, *T*; *v*) ≡ 𝓔(D), (70)

while the latter corresponds to the quantum expectation of (Hermitian) operator for the resultant gradient information of *N_i_* electrons, {I*_i_* ≡ I(*N_i_*) ≡ ∑*_k_* I(*k*)}, related to the corresponding kinetic-energy operators {T*_i_* ≡ T(*N_i_*) = ∑*_k_* T(*k*)}, *k* = 1, 2, …, *N_i_*,

I*_i_* = −4∑*_k_* Δ*_k_* ≡ ∑*_k_* I(*k*) = (8*m*/*ħ*^2^) T*_i_* ≡ *κ* T*_i_* = *κ* ∑*_k_* T(*k*), T(*k*) = −[*ħ*^2^/(2*m*)] Δ*_k_*, (71)

〈*I*〉*_ens._* = ∑*_i_*∑*_j_P_j_^i^*(*μ*, *T*; *v*) 〈Ψ*_j_^i^*|I*_i_*|Ψ*_j_^i^*〉 ≡ ∑*_i_*∑*_j_P_j_^i^*(*μ*, *T*; *v*) *I_j_^i^* ≡ *𝓘*(*μ*, *T*; *v*) ≡ *𝓘*(D), (72)

Thus the average gradient information *𝓘*(D) reflects the (dimensionless) average kinetic energy

〈*T*〉*_ens._* = ∑*_i_* ∑*_j_ P_j_^i^*(*μ*, *T*; *v*) 〈Ψ*_j_^i^*|T*_i_*|Ψ*_j_^i^*〉 ≡ ∑*_i_*∑*_j_ P_j_^i^*(*μ*, *T*; *v*) *T_j_^i^* ≡ 𝓣(*μ*, *T*; *v*) = 𝓣(D) = *κ*^−1^*𝓘*(D),

*T_j_^i^* = 〈Ψ*_j_^i^*|T*_i_*|Ψ*_j_^i^*〉 = *κ*^−1^*I_j_^i^*. (73)

The equilibrium probabilities **P**(*μ*, *T*; *v*) result from the minimum principle of the *grand* potential *Ω*(D):

*δ*[𝓔(D) − *μ* 𝓝(D) − *T**Տ***(D)]|**_P_**_(*μ*,*T*; *v*)_ ≡ *δΩ*(D)|**_P_**_(*μ*,*T*; *v*)_ = 0. (74)

Here, the average number of electrons

〈*N*〉*_ens._* = ∑*_i_N_i_* [∑*_j_P_j_^i^*(*μ*, *T*; *v*)] ≡ ∑*_i_N_i_ P^i^*(*μ*, *T*; *v*) = 𝓝(D) (75)

and the thermodynamic entropy of the ensemble

〈*S*〉*_ens._* = −*k*_B_ ∑*_i_*∑*_j_ P_j_^i^*(*μ*, *T*; *v*) ln*P_j_^i^*(*μ*, *T*; *v*) ≡ ***Տ***(D), (76)

with *k*_B_ denoting the Boltzmann constant.

The entropy-constrained *energy* principle of Equation (74) can be also interpreted as an equivalent (potential-energy constrained) *information* rule [5,6,9,67,68,69], for the minimum of the ensemble resultant gradient-information *𝓘*(D):

*δ*[*𝓘*(D) − *λ* 𝓦(D) − *ζ* 𝓝(D) − *τ**Տ***(D)]**_P_**_(_*_μ_*_,*T*; *v*)_ = 0. (77)

It contains the additional constraint of the fixed overall potential energy, 〈*W*〉*_ens._* = 𝓦(D), multiplied by the Lagrange multiplier *λ* = −*κ*, and includes the “scaled” *information* intensities associated with the remaining constraints:

*potential ζ* = *κ μ*, enforcing the prescribed electron population 𝓝(D) = *N*; 

*temperature τ* ≡ *κ T*, for the subsidiary entropy condition, ***Տ***(D) = *S*.

The extrema of the ensemble principles of Equations (74) and (77) determine the same equilibrium probabilities **P**(*μ*, *T*; *v*) of electronic states. The physical equivalence of the energy and information principles indicates that energetic and information reactivity concepts are mutually related, being both capable of describing charge-transfer (CT) phenomena in acid(A)–base(B) systems.

The ensemble interpretation applies to all populational, 𝓝-derivatives of the average energy or information functionals. For example, in energy representation the global chemical hardness [44,63] reflects the 𝓝-derivative of the chemical potential,

*η* = ∂^2^𝓔/∂𝓝^2^ = ∂*μ*/∂𝓝 > 0, (78)

while the information hardness measures the 𝓝-derivative of the information potential:

*ω* = ∂^2^*𝓘*/∂𝓝^2^ = ∂*ζ*/∂𝓝 = *κ η* > 0. (79)

The positive signs of these “diagonal” (hardness) derivatives assure the external stability of 〈M(*v*)〉*_ens._*, with respect to charge flows between the molecular system M(*v*) and its electron reservoir, in accordance with the Le Châtelier and Le Châtelier–Braun principles of thermodynamics [70].

The global FF [44,56,57,58,59,64] is defined by the “mixed” second derivative of the ensemble average energy:

*f*(***r***) = ∂/∂𝓝[*δ*𝓔/*δv*(***r***)] = ∂*ρ*(***r***)/∂𝓝 = *δ*/*δv*(***r***) (∂𝓔/∂𝓝) = *δμ*/*δv*(***r***), (80)

where we have applied the Maxwell cross-differentiation identity. It can be thus interpreted as either the density response per unit populational displacement, or as the response in the global chemical potential to unit displacement in the local external potential. The analogous derivative of the average gradient information similarly reads:

*ϕ*(***r***) = ∂/∂𝓝[*δ*𝓘/*δv*(***r***)] = *δ*/*δv*(***r***) (∂𝓘/∂𝓝) = *κ f*(***r***) = *δζ*/*δv*(***r***). (81)

The in situ CT derivatives of the average resultant gradient information in the reactive system R = A–B include the CT potential quantity, related to *μ*_CT_,

*ζ*_CT_ = ∂𝓘(*N*_CT_)/∂*N*_CT_ = *κ μ*_CT_, (82)

and the CT hardness descriptor, related to *η*_CT_ = *S*_CT_^−1^,

*ω*_CT_ = ∂^2^𝓘(*N*_CT_)/∂*N*_CT_^2^ = ∂*ζ*(*N*_CT_)/∂*N*_CT_ = *κ η*_CT_ ≡ *θ*_CT_^−1^, (83)

which is the inverse of the CT softness *θ*_CT_ = ∂*N*_CT_/∂*ζ*. In terms of these CT descriptors, the optimum amount of the B→A electron transfer in the donor–acceptor reactive system,

*N*_CT_ = 𝓝_A_ − *N*_A_^0^ = *N*_B_^0^ − 𝓝_B_ > 0,

thus reads:

*N*_CT_ = − *μ*_CT_/*η*_CT_ = −*μ*_CT_*S*_CT_ = − *ζ*_CT_/*ω*_CT_ = −*ζ*_CT_*θ*_CT_. (84)

Above, {*N*_X_^0^} and {𝓝_X_} denote electron populations of the mutually *closed* and *open* reactants in M^+^ = (A^+^|B^+^) and M^*^ = (A^*^¦B^*^) = M, respectively. 

Therefore, the in situ derivatives {*ζ*_CT_, *ω*_CT_ = *θ*_CT_^−1^} of the average content of the resultant gradient information provide alternative reactivity descriptors, equivalent to the chemical potential and hardness or softness indices {*μ*_CT_, *η*_CT_ = *S*_CT_^−1^} of the classical, energy-centered theory of chemical reactivity. This again demonstrates the physical equivalence of the energy and information principles in describing the CT phenomena in molecular systems. One thus concludes that the resultant gradient information, the quantum generalization of the classical Fisher measure, constitutes a reliable basis for an “entropic” description of reactivity phenomena.

## 7. Latent Probability Flows in Stationary Equilibrium

Consider again the stationary state *ψ_st._*(***r***, *t*) of an electron (Equation (6)) corresponding to the sharply specified energy *E_st._*. The wavefunction phase is then purely time dependent, *φ_st._*(***r***, *t*) = −*ω_st._t* ≡ *φ_st._*(*t*), with the state local aspect being described solely by its modulus part *R_st._*(***r***), the eigenfunction (see Equation (9)) of the electronic Hamiltonian of Equation (4). This stationary “equilibrium” thus generates the vanishing probability current ***j****_st._*(***r***) = *p_st._*(***r***)***V****_st._*(***r***), where the time-independent probability distribution *p_st._*(***r***) = *R_st._*(***r***)^2^ and the vanishing flux-velocity ***V****_st._*(***r***) = (*ħ*/*m*) ∇*φ_st._*(*t*) = ***0***. As indicated in Section 2, the eigenstates of the electronic Hamiltonian correspond to the equalized local energy, *E_st._*(***r***,*t*) = *E_st._*, marking the equalized local phase: *φ_st._*(***r***, *t*) = *φ_st._*(*t*).

Clearly, the stationary probability distribution and its vanishing current/velocity in such states do not imply that the particle is then at rest. The electrons are incessantly moving around the fixed nuclei, with the experimentally (sharply) unobserved instantaneous *particle* velocity ***W***(***r***, *t*) = *d**r***(*t*)/*dt* = ***P***(***r***, *t*)/*m* reflecting its momentum ***P***(***r***, *t*) = *ħ **k***(***r***, *t*). Indeed, the system stability requires that centrifugal forces of these fast movements compensate for the nuclear attraction as, e.g., in Bohr’s historic, “planetary” model of the hydrogen atom. The tightly bound *inner* (“core”) electrons have to move faster than less confined *outer* (“valence”) electrons. The natural question then arises: how to describe the presence of these unceasing (latent) instantaneous motions in the dynamics of the probability “fluid”?

**Figure 2:**
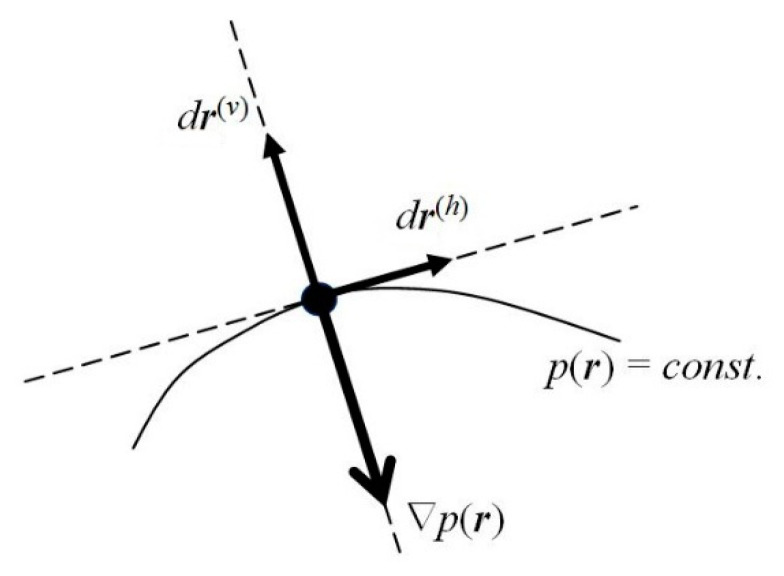
Local “vertical” (*v*) and “horizontal” (*h*) directions.

**Figure 3:**
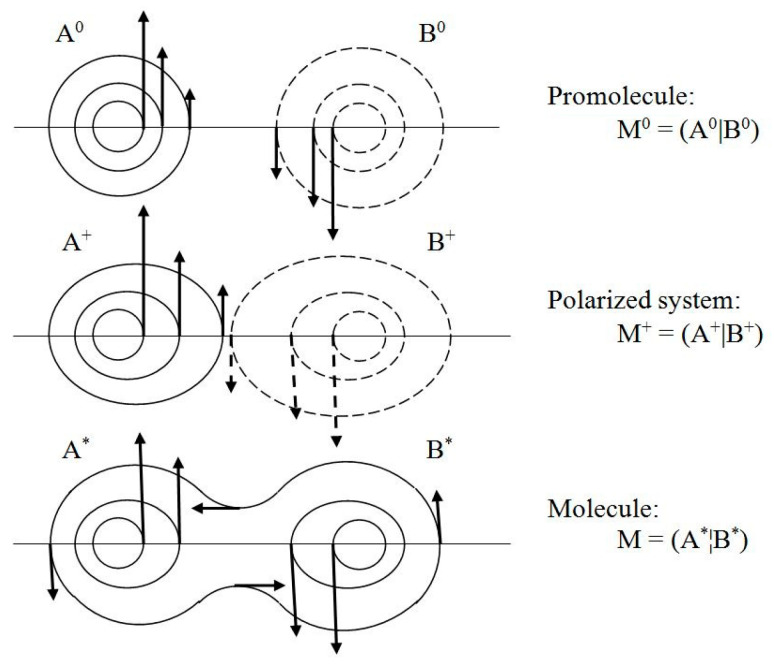
Schematic diagrams of atomic and molecular vortices of “horizontal” flows of electronic probability density in atomic fragments of diatomic promolecule M^0^, the polarized system M^+^, and in molecule M.

One observes that, for the probability density *p_st._*(***r***) to remain conserved in time, its latent flows must follow the probability contours *p_st._*(***r***) = *p*^(0)^ = *const*. (see Figure 2). Any motion in the direction perpendicular to the probability line passing a given location in space would imply a change in time of the probability value at this point, and hence the nonstationary character of the whole distribution. In other words, the latent flows of the stationary position-probability distribution must be “horizontal”, directed along the constant-probability lines. Such probability fluxes in *ψ_st._*, along the probability contours for the vanishing “vertical” velocity component, preserve in time the stationary character of the spatial probability distribution, which determines the vanishing probability flux. Therefore, the stationary character of the molecular electronic state does not preclude the latent local flows of electronic probability in horizontal directions generating the atomic vortices of Figure 3.

The instantaneous *resultant* (*r*) velocity ***V***^(*r*)^(***r***, *t*) of probability “fluid” thus involves two independent components (see Figure 2): the “*vertical*” (current) velocity along the phase gradient,

***V***^(*v*)^(***r***, *t*) ≡ ***V***(***r***, *t*) = ***j***(***r***, *t*)***/****p*(***r***, *t*) = (*ħ*/*m*) ∇*φ*(***r***, *t*), (85)

perpendicular to the local direction of probability contour at time *t*, ***V*** ⊥ (*p* = *const.*), and hence parallel to ∇*p*(***r***, *t*), ***V***||(∇*φ*, ∇*p*); and the “*horizontal*” velocity ***V***^(*h*)^(***r***, *t*), along the probability contour,

***V***^(*r*)^(***r***, *t*) = ***V***(***r***, *t*) +***V***^(*h*)^(***r***, *t*). (86)

The horizontal velocity ***V***^(*h*)^(***r***, *t*) of probability motions along the constant-probability lines, ***V***^(*h*)^||(*p* = *const.*), can also remain finite in the stationary electronic states of atomic or molecular systems, since it does not affect the conserved probability distribution. The vertical component ***V*** of the probability current then reflects a common direction of gradients ∇*φ* and ∇*p*, of the distributions’ fastest increase, with a horizontal supplement perpendicular to both these gradients: ***V***^(*h*)^⊥(∇*φ*, ∇*p*).

These components of probability velocity imply the associated combination rules for the resultant probability and phase currents:

***j***^(*r*)^(***r***, *t*) = *p*(***r***, *t*) ***V***^(*r*)^(***r***, *t*) = *p*(***r***, *t*)***V***(***r***, *t*) + *p*(***r***, *t*)***V***^(*h*)^(***r***, *t*) ≡ ***j***(***r***, *t*) +***j***^(*h*)^(***r***, *t*), (87)

***J***^(*r*)^(***r***, *t*) = *φ*(***r***, *t*)***V***^(*r*)^(***r***, *t*) = *φ*(***r***, *t*)***V***(***r***, *t*) + *φ*(***r***, *t*)***V***^(*h*)^(***r***, *t*) ≡ ***J***(***r***, *t*) +***J***^(*h*)^(***r***, *t*). (88)

The above directional properties of the vertical and horizontal components then confirm the validity of the (vertical) continuity relations for the probability and phase distributions:

∂*p*/∂*t* = −**∇⋅*j***^(*r*)^ = −**∇***p*⋅***V***^(*r*)^ = −∇*p***⋅**[***V*** + ***V***^(*h*)^] = −∇*p***⋅*V*** = −∇**⋅*j***, (89)

∂*φ*/∂*t* = −**∇⋅*J***^(*r*)^ + *σ_φ_* = −∇*φ***⋅*V***^(*r*)^ + *σ_φ_* = −∇*φ***⋅** [***V*** + ***V***^(*h*)^] + *σ_φ_* = −∇*φ***⋅*V*** + *σ_φ_* = −∇**⋅*J*** + *σ_φ_*, (90)

where we have recognized Equation (55) and observed that horizontal currents ***j***^(*h*)^ and ***J***^(*h*)^ generate vanishing divergences,

**∇⋅*j***^(*h*)^ = ∇*p***⋅*V***^(*h*)^ = 0 and **∇⋅*J***^(*h*)^ = ∇*φ***⋅*V***^(*h*)^ = 0, (91)

since ***V***^(*h*)^ is perpendicular with respect to both ∇*p* and ∇*φ*.

Therefore, the phase and probability gradients are both perpendicular to the probability contour and, hence, ∇*φ*(***r***, *t*) ∝ ∇*p*(***r***, *t*). This directional character of the current velocity ***V***(***r***, *t*) suggests that the local aspect of the phase function itself should be related to the probability density:

*φ*(***r***, *t*) = *φ*[*p*(***r***, *t*), *t*] ⇒ ∇*φ* = (∂*φ*/∂*p*) ∇*p*. (92)

Such a directional feature indeed characterizes the IT equilibrium (“thermodynamic”) phase of Equation (32) (see also Section 4), resulting from extrema of the phase entropy/information functionals,

*φ_eq._*(***r***, *t*) = −(1/2) ln*p*(***r***, *t*) ≥ 0, (93)

for which

∇*φ_eq._*(***r***, *t*) = −[2*p*(***r***, *t*)]^−1^ ∇*p*(***r***, *t*). (94)

The velocity of the latent, “horizontal” flows along the probability contours can be then attributed to the additional (local) *horizontal* phase *φ*^(*h*)^(***r***) component, a “thermodynamic” addition to the purely time-dependent stationary phase *φ_st._*(*t*) in the resultant phase of the transformed state:

Φ(***r***, *t*) = *φ_st._*(*t*) + *φ*^(*h*)^(***r***), (95)

***V***^(*r*)^(***r***, *t*) = (*ħ*/*m*) ∇Φ(***r***, *t*) = (*ħ*/*m*) ∇*φ*^(*h*)^(***r***) = ***V***^(*h*)^(***r***). (96)

In order to study the time-dependent flows in liquids, the separate concepts of “streamline” and “pathline” are introduced [71]. At the specified time, the former are tangential to the directional field of velocity “arrows”. Since the particles move in the direction of the streamlines, there is no motion perpendicular to the streamlines and the property flux per unit time between two streamlines remains constant. Patterns of streamlines describe the instantaneous state of a flow, indicating the direction of motion of all particles at a given time. For the time-dependent flows, the velocity field changes in time, with pathlines no longer coinciding with streamlines. Only for the time-independent flows do the particles move along streamlines, so that pathlines and streamlines coincide.

In the stationary quantum mechanics the contours of molecular probability “fluid” at time *t* = *t*_0_, *p*(***r***, *t*_0_) = *p_st._*(***r***) similarly determine the streamlines of the latent (horizontal) flows of electronic probability, which preserve the “static” probability distribution *p_st._*(***r***) of the stationary quantum state. They generate “vortices” of the latent “horizontal” velocity in spherical probability distributions of free atoms of the promolecule M^0^, the deformed AIM distributions in the polarized system M^+^, and in the equilibrium density of the molecule M = M* (see Figure 3).

## 8. Component Dynamics in Equilibrium Stationary States

Consider again a general (complex) state of an electron (Equation (1)) and its quantum dynamics in Equation (5), determined by the Hamiltonian of Equation (4). Let us separate the local “vertical” ***r***^(*v*)^ and “horizontal” ***r***^(*h*)^ components of a general displacement in electronic position (see Figure 2), *d**r*** = *d**r***^(*v*)^ + *d**r***^(*h*)^, in directions perpendicular and parallel to the probability contour *p*(***r***) = *const.*, respectively. The former is consistent with the probability gradient ∇ *p*(***r***), which reflects the direction of the distribution fastest increase.

The stationary (ground) state of an electron *ψ*_0_, for the sharply specified energy,

*E*[*ψ*_0_] = 〈*ψ*_0_|H|*ψ*_0_〉 = 〈*R*_0_|H|*R*_0_〉 = *E*_0_ ≡ *E_v_*[*p*_0_], (97)

corresponds to the time-independent modulus function *R*_0_(***r***) and time-dependent phase component *φ*_0_(*t*) = −(*E*_0_/*ħ*)*t* ≡ *−ω*_0_ *t*. The associated equilibrium state then corresponds to the locally (horizontally) modified resultant phase,

Φ(***r***, *t*) = *φ*_0_(*t*) + *φ*^(*h*)^(***r***), (98)

in the *phase*-transformed wavefunction,

Ψ_0_(***r***, *t*) = *ψ*_0_(***r***, *t*) exp[i*φ*^(*h*)^(***r***)] = *R*_0_(***r***) exp{i[*− ω*_0_*t* + *φ*^(*h*)^(***r***)]} ≡ *R*_0_(***r***) exp{iΦ(***r***, *t*)]} ≡ *ϕ*_0_(***r***) exp{i*φ*_0_(*t*)]}, (99)

which conserves the stationary probability distribution:

*p*_0_(***r***) = |Ψ_0_(***r***, *t*)|^2^ = |*ψ*_0_(***r***)|^2^ = |*ϕ*_0_(***r***)|^2^ = [*R*_0_(***r***)]^2^. (100)

However, the expectation value of the energy,

*E*[Ψ_0_] = 〈Ψ_0_|H|Ψ_0_〉 = 〈*ϕ*_0_|H|*ϕ*_0_〉 = *E*_0_ + *T*[*φ*^(*h*)^], (101)

differs from *E*_0_ of Equation (97) by the “horizontal” kinetic energy,

*T*[*φ*^(*h*)^] = *κ*^−1^*I*[*φ*^(*h*)^] = ∫*p*_0_(***r***){(*m*/2)[(*ħ*/*m*)∇*φ*^(*h*)^(***r***)]^2^*d**r*** ≡ ∫*p*_0_(***r***){(*m*[***V***^(*h*)^(***r***)]^2^/2)}*d**r*** ≡ ∫*p*_0_(***r***) *T*^(*h*)^(***r***) *d**r***, (102)

related to the (horizontal) nonclassical information,

*I*[*φ*^(*h*)^] = 4∫*p*_0_(***r***) [∇*φ*^(*h*)^(***r***)]^2^*d**r***.

The normalization-constrained minimum principle for this average energy gives the following stationary SE, including the horizontal kinetic-energy contribution:

*δ*{*E*[Ψ] − *λ* 〈Ψ|Ψ〉}|_0_ = 0 or

HΨ_0_ = {*E*_0_ + *T*[*φ*^(*h*)^]}Ψ_0_ ≡ *ħ ω*[Ψ_0_] Ψ _0_ = *ħ* {*ω*_0_ + *T*[*φ*^(*h*)^]/*ħ*}Ψ_0_ ≡ *ħ* {*ω*_0_ + *ω*^(*h*)^}Ψ_0_. (103)

This *horizontally*-generalized stationary SE thus includes the additional wave-number contribution *ω*^(*h*)^ = *T*[*φ*^(*h*)^]/*ħ*. The DFT minimum principle of *E_v_*[*p*], equivalent to the ordinary (stationary) SE,

H*R*_0_ = {−[*ħ*^2^/(2*m*)]Δ + *v*} *R*_0_ = *E*_0_*R*_0_ = *ħω*_0_*R*_0_, (104)

determines the optimum probability distribution, *p_opt._* = *p*_0_ = *R*_0_^2^, and energy *E_opt._* = *E_v_*[*p*_0_] = *E*_0_, while the equilibrium horizontal (“thermodynamic”) phase is determined by a supplementary IT rule (see Section 3).

In the stationary equilibrium,

*∂R*_0_/∂*t* = *∂φ*^(*h*)^/∂*t* = 0 and ∇Φ = ∇*φ*^(*h*)^, (105)

and the horizontal velocity of probability flux reflects the gradient of *φ*^(*h*)^:

***V***^(*h*)^ = *d**r***^(*h*)^/*dt* = (*ħ*/*m*) ∇*φ*^(*h*)^. (106)

The resultant probability velocity is then exclusively of a horizontal origin,

***V***_0_ = (*ħ*/*m*)∇Φ = (*ħ*/*m*)∇*φ*^(*h*)^ ≡ ***V***^(*h*)^, (107)

and both components of Φ contribute to the resultant *phase* source in the associated continuity equation:

*σ*_Φ_ = *d*Φ/*dt* = *dφ*_0_/*dt* + *dφ*^(*h*)^/*dt* ≡ *σ*_0_ + *σ_h_* = *σ*_0_ + ***V***^(*h*)^ ⋅∇*φ*^(*h*)^ = *σ*_0_ + ∇⋅***J***^(*h*)^. (108)

Therefore, in the stationary equilibrium, the *vertical* source of the wavefunction phase remains constant, *σ*_0_ = *dφ*_0_/*dt* = *−ω*_0_, while the local horizontal-phase source assumes a purely convectional character:

*σ_h_* ≡ *dφ*^(*h*)^/*dt* = [*d**r***^(*h*)^/*dt*] [∂*φ*^(*h*)^/∂***r***^(*h*)^]= ***V***^(*h*)^ ⋅∇*φ*^(*h*)^ = ∇⋅***J***^(*h*)^. (109)

The SE for components of Ψ_0_ reads:

*∂*Φ/∂*t* = *∂φ*_0_/∂*t* = *−ω*_0_ = *ħ*(2*m*)^−1^{*R*_0_^−1^Δ*R*_0_ + 2i∇*R*_0_⋅∇*φ*^(*h*)^ − [∇*φ*^(*h*)^]^2^} − *v*/*ħ*. (110)

Its imaginary part confirms that ***V***^(*h*)^ = (*ħ*/*m*)∇*φ*^(*h*)^, and hence also the associated probability current, ***j***^(*h*)^ = *p*_0_⋅***V***^(*h*)^, are indeed perpendicular to the probability gradient ∇*p*_0_ = 2*R*_0_∇*R*_0_,

∇*p*_0_**⋅*V***^(*h*)^ = ∇⋅***j***^(*h*)^ = 0. (111)

The real part of Equation (110) generates the associated phase dynamics,

*∂*Φ/∂*t* = *−ω*_0_ = *ħ*(2*m*)^−1^{*R*_0_^−1^Δ*R*_0_ − [∇*φ*^(*h*)^]^2^} − *v*/*ħ =* −∇⋅***J***^(*h*)^ + *σ*_0_, (112)

where the horizontal phase current ***J***^(*h*)^ = *φ*^(*h*)^***V***^(*h*)^, ∇⋅***J***^(*h*)^ = ∇*φ*^(*h*)^⋅***V***^(*h*)^ and the resultant phase source is defined in Equation (108).

## 9. Conclusions

In this conceptual overview we have first examined the spatial equalization of the electronic phase in molecules, using the (complex) local-energy concept. The real component of *E*(***r***, *t*) was shown to shape the dynamics of wavefunction phase, while the time evolution of the state modulus was shown to be governed by the imaginary component of local energy. In QM the spatial equalization of the local wave-number or phase concepts marks the system stationary state. The resultant IT descriptors, combining the modulus/probability and phase/current contributions, were revisited and the wave-function mapping in QM was compared with the probability scheme of classical IT. The nonclassical, phase/current supplements in the resultant IT measures effectively lower the classical entropic uncertainty and increase the spatial information determinicity of quantum states.

The *phase*-transformed (“thermodynamic”) states were introduced and their IT optimum phases were determined from the auxiliary entropic principle. These equilibrium states were shown to exhibit the exactly vanishing *internal* (resultant) IT descriptors of electronic states. Therefore, in statistical mixtures of such states, the overall entropy content reduces to the *external* ensemble entropy of von Neumann. This brings more consistency into the quantum IT description of *open* molecular systems. We have also summarized the local continuity relations for the wavefunction components, state physical descriptors, and information densities. They follow from the component-resolved SE and the realization that flows of molecular properties are carried by (convection) fluxes of electronic probability. Therefore, in such treatments, the electrons are carriers of densities of both the system physical and information properties.

The principal variational principle for the minimum of the *grand* potential was interpreted as an equivalent information rule. In an ensemble description of chemical reactions in the acid–base systems, the populational derivatives of the ensemble-average resultant information were shown to constitute adequate entropic criteria for diagnosing the molecular CT phenomena, fully equivalent to their energy analogs. Latent electronic fluxes in the stationary molecular states were identified. These hidden (“horizontal”) electronic flows, along the constant-probability contours, do not affect the stationary probability distribution and generate velocity vortices in molecules. Using the SE for wavefunction components, their local velocity was related to the “thermodynamic” phase of the phase-transformed equilibrium states.

## Figures and Tables

**Table 1:** Summary of wavefunction components of the quantum state |*ψ*(*t*)〉 of an electron, their dynamics, physical descriptors and local sources.

Schrödinger equation:	H |*ψ*(*t*)〉 = i*ħ* [∂|*ψ*(*t*)〉/∂*t*]
Wavefunction:	*ψ*[***r***(*t*), *t*] = 〈***r***(*t*)|*ψ*(*t*)〉 ≡ *ψ*(***r***, *t*) = *R*(***r***, *t*) exp[i*φ*(***r***, *t*)]
modulus	*R*(***r***, *t*), ∂*R*(***r***, *t*)/∂*t* = −***V***(***r***, *t*)⋅∇*R*(***r***, *t*)
phase	*φ*(***r***, *t*), ∂*φ*(***r***, *t*)/∂*t* = *ħ*(2*m*)^−1^ {*R*(***r***, *t*)^−1^ Δ*R*(***r***, *t*) − [∇*φ*(***r***, *t*)]^2^} − *v*(***r***)/*ħ*
time-dependence	Explicit, due to |*ψ*(*t*)〉, and implicit, due to |***r***(*t*)〉
logarithm	ln*ψ*(***r***, *t*) = ln*R*(***r***, *t*) + i*φ*(***r***, *t*) = ½ ln*p*(***r***, *t*) + i*φ*(***r***, *t*)
Descriptors of electron probability density *p*(***r***, *t*) = *R*(***r***, *t*)^2^:	
current	***j***(***r***, *t*) = (*ħ*/*m*) *p*(***r***, *t*) ∇*φ*(***r***, *t*) = *p*(***r***, *t*) ***V***(***r***, *t*)
velocity	***V***(***r***, *t*) ≡ ***j***(***r***, *t*)/*p*(***r***, *t*), ∇⋅***V***(***r***, *t*) = (*ħ*/*m*)Δ*φ*(***r***, *t*) = 0
acceleration	***a***(***r***, *t*) = *d**V***(***r***, *t*)/*dt* = (*ħ*/*m*)∇*σ_φ_*(***r***, *t*)
force	***F***(***r***, *t*) = *m **a***(***r***, *t*) ≡ −∇*W*(***r***, *t*)
potential	*W*(***r***, *t*) = − ∫***F***(***r***, *t*) *d**r*** = −*ħσ_φ_*(***r***, *t*)
Resultant gradient information:	*I*[*ψ*] = ∫*p*(***r***, *t*){[∇ln*p*(***r***, *t*)]^2^ + 4 [∇*φ*(***r***, *t*)]^2^} *d**r*** ≡ ∫*p*(***r***, *t*)*I*(***r***, *t*) *d**r***
Convection operator:	***V***(***r***, *t*)⋅∇ = *d*/*dt* − ∂/∂*t*
Sources: probability	*σ_p_*(***r***, *t*) = *dp*(***r***, *t*)/*dt* = ∂*p*(***r***, *t*)/∂*t* + ∇⋅ ***j***(***r***, *t*) = 0
phase	*σ_φ_*(***r***, *t*) = *dφ*(***r***, *t*)/*dt* = ∂*φ*(***r***, *t*)/∂*t* + ∇⋅***J***(***r***, *t*) = *ħ*(2*m*)^−1^ {*R*(***r***, *t*)^−1^Δ*R*(***r***, *t*) + [∇*φ*(***r***, *t*)]^2^} − *v*(***r***)/*ħ* ***J***(***r***, *t*) = *φ*(***r***, *t*) ***V***(***r***, *t*)
current	*σ**_j_***(***r***, *t*) = *d**j***(***r***, *t*)/*dt* = *σ_φ_*(***r***, *t*)***V***(***r***, *t*) + *φ*(***r***, *t*) ***a***(***r***, *t*)
information	*σ_I_*(*t*) = *κ*∫***j***(***r***, *t*)⋅∇*v*(***r***) *d**r*** = *κ ħ* ∫***j***(***r***, *t*)⋅∇*σ_φ_*(***r***, *t*) *d**r*** *κ* = 8*m*/*ħ*^2^

## Data Availability

Not applicable.
